# Association of MBI‐C scores with clinical outomes in patients with MCI during a 3‐year follow‐up

**DOI:** 10.1002/alz70857_099634

**Published:** 2025-12-24

**Authors:** Nikita Cherkasov, Igor Kolykhalov

**Affiliations:** ^1^ Mental Health Research Center, Moscow, Moskva, Russian Federation

## Abstract

**Background:**

As previously shown, mild behavioral impairment (MBI), characterized by late‐life emergent and persistant neuropsychiatric symptoms (NPS) with minimal impact on daily life, is associated with increased dementia risk. Z. Ismail et al. have proposed an informant‐based checklist (MBI‐C), a 34‐item list, structured into 5 domains in accordance with ISTAART criteria, to quantify MBI. This study examines the MBI‐C utility in Russian‐speaking patients with mild cognitive impairment (MCI).

**Method:**

Eighty‐six patients (age 71 [65;76] years, 70 female) with MCI (according to R. Petersen criteria) underwent a clinical, neuropsychological, and behavioral assessment in a specialized Alzheimer's disease department, with a 3‐year follow‐up. Montreal Cognitive Assessment (MoCA), Clinical Dementia Rating (CDR), and MBI‐C scores were obtained at baseline and follow‐up visits. MBI was diagnosed based on ISTAART criteria and with an MBI‐C total score of >6. A diagnosis of dementia was based on ICD‐10 criteria and CDR total score of >1. Statistical analysis included the Wilcoxon signed‐rank test to evaluate differences between two time points, a two‐way ANOVA was performed to evaluate the effect of the MBI‐C total score over time on conversion to dementia.

**Result:**

Eighteen patients converted to dementia (16‐AD, 2‐bvFTD). Converters had significantly higher baseline MBI‐C scores (8 [6;15.5]) compared to non‐converters (4 [1;10], *p* = 0.002). Differences were also significant in all domains except affective dysregulation. Pairwise comparisons showed that the mean MBI‐C scores in impulse dyscontrol (*p* = 0.03) and in total (*p* = 0.009) increased significantly in converters over time. A significant interaction between baseline MBI‐C total score and clinical outcome was found (F(1,164) = 56.6, *p* <0.001) with a large effect size (ges=0.26). While time alone had no effect on MBI‐C and conversion, patients with MCI and MBI showed significant MoCA decline (22.6 to 20.6, *p* = 0.007), unlike MCI‐only patients (24.2 to 24.3).

**Conclusion:**

The MBI‐C shows strong utility in a Russian‐speaking MCI population. It demonstrates relevance as a sensitive measure of behavioral symptoms that precede dementia. The strong association between baseline MBI‐C scores and subsequent decline highlights its predictive power for the progression of neurodegenerative disorders, complementing routine cognitive measures.

